# Highly Differentiated Follicular Carcinoma of Ovarian Origin: A Systematic Review of the Literature

**DOI:** 10.3390/curroncol29120712

**Published:** 2022-11-23

**Authors:** Eirini Giovannopoulou, Konstantinos Saliaris, Evangelia Kavoura, Kitty Pavlakis, Konstantinos Lathouras

**Affiliations:** 1 Department of Gynecological Oncology, IASO Hospital, 151 23 Athens, Greece; 2 Pathology Department, IASO Hospital, 151 23 Athens, Greece

**Keywords:** struma ovarii, ovarian malignancy, thyroid cancer

## Abstract

(1) Background: Highly differentiated follicular carcinoma of ovarian origin (HDFCO) is an extremely uncommon neoplasm, associated with struma ovarii. There are scarce cases reported in the literature and, subsequently, no reliable conclusions on its pathophysiology, treatment, and prognosis can be drawn. The goal of this study is to enrich the literature on the topic by adding our own experience with a case, and simultaneously accumulate all cases published up to date. (2) Methods: The present review was performed in accordance with the guidelines for systematic reviews and meta-analyses (PRISMA). PubMed (1966–2022), Scopus (2004–2022), and Clinicaltrials.gov databases were screened for relevant articles published up to July 2022. (3) Results: Twenty patients with HDFCO were identified. The included patients were aged 47.15 years (range 24–74). The predominant origin was ovarian (60%) and extraperitoneal spread was confirmed in 15% of the cases. Surgical treatment varied from conservative to radical (35.3% vs. 41.2%, respectively) and the administration of supplementary therapy and thyroidectomy was not universal. Combined thyroidectomy/radioactive iodine therapy was applied in just 62.5% of the reported cases. There was one patient who demonstrated disease recurrence and lives with the disease. No disease related morbidity was reported. (4) Conclusions: HDFCO represents a low-grade malignant tumor, whose rarity does not allow for reliable conclusions. Standard treatment including complete surgical excision and supplementary treatment seems to offer a favorable prognosis in selected cases.

## 1. Introduction

Struma ovarii is a rare monodermal tumor of the ovaries, comprised mainly of thyroid tissue [1]. In order to qualify as a struma ovarii, an ovarian teratoma must be composed of at least 50% mature thyroid tissue [2]. Struma ovarii represents 1% of all ovarian tumors and 2.7% of dermoid tumors [3]. Although the majority of struma ovarii are benign, as many as 5–10% of them are proven histologically malignant, with papillary thyroid carcinoma being the most commonly identified histological subtype [4,5,6].

Nomenclature regarding struma ovarii subtypes was recently updated by the WHO, in light of concerns about the possible malignant potential of tumors comprised of thyroid tissue without histologic neoplastic features identified in extra-ovarian sites [7]. Until recently, all these cases were collectively referred to as “peritoneal strumosis” or “struma peritonei” in the literature, due to the absence of histological features of malignancy and despite their biologic behavior with a tendency to spread and recur [8]. In the 2020 WHO Classification of Female Genital Tumors, an extremely rare histological subtype called highly differentiated follicular carcinoma arising from struma ovarii (HDFCO) was introduced, representing the entity formerly described as peritoneal strumosis, in order to highlight its low-grade malignant potential [7]. The term was initially introduced by Roth et al. in 2008, in an attempt to reexamine the cases collectively referred to as “peritoneal strumosis”. Due to its mature histological appearance, HDFCO is only diagnosed when signs of spread beyond the ovary are exhibited, proving its malignant behaviour [7].

This emerging clinical entity is extremely rare, with only few cases published in the literature. The treatment most commonly involves local excision of the tumor and thyroidectomy followed by high-dose radio iodine therapy, in a similar fashion as for metastatic thyroid carcinoma and malignant struma ovarii [2,9,10,11,12,13,14]. However, the safety, efficacy, and oncologic outcome of these interventions have not been validated [2,4,7,8,9,10,11,12,13,14,15].

This review aims to accumulate the current knowledge on highly differentiated struma of ovarian origin (HDFCO), specially commenting on the management and the oncologic outcomes. Along with the cases derived from the literature, we present an additional case of a 25-year-old woman with HDFCO that was effectively treated with uterine-sparing treatment in our department.

## 2. Materials and Methods

The present review was performed in accordance with the guidelines for systematic reviews and meta-analyses (PRISMA), based on the authors’ pre-determined inclusion criteria [16]. The literature was searched by two independent reviewers and eligible studies were selected.

### 2.1. Data Sources and Search Strategy

A systematic search of the PubMed (1966–2022), Scopus (2004–2022), and Clinicaltrials.gov databases for articles published up to July 2022 was performed under a common standardized search protocol with the following combination of keywords: highly differentiated follicular carcinoma of ovarian origin OR highly differentiated follicular carcinoma in ovary OR peritoneal strumosis OR struma peritonei OR extremely well differentiated follicular carcinoma of ovarian origin.

Considering the characteristics of the sample, the following quantitative variables were evaluated: age, size of the lesion, and follow-up period. The measured qualitative variables were: presenting complaints, previous gynecologic surgery or any previous surgery associated with struma ovarii, location of the lesions, presence of ascites, type of surgery, the use of adjuvant therapy, and recurrences.

### 2.2. Eligibility Criteria

Articles written in the English language and available in full-text were assessed by the reviewers. Due to the extreme rarity of the investigated entity and the absence of prospective or retrospective studies, data were solely derived from case reports and case series. Articles that presented cases of HDFCO and offered relevant data on their management and patients’ outcomes were considered eligible for inclusion. Reviews were excluded from the analysis, except form studies that included case reports with sufficient documentation, which were independently included in the analysis.

## 3. Results

### 3.1. Included and Excluded Studies

**Figure 1:**
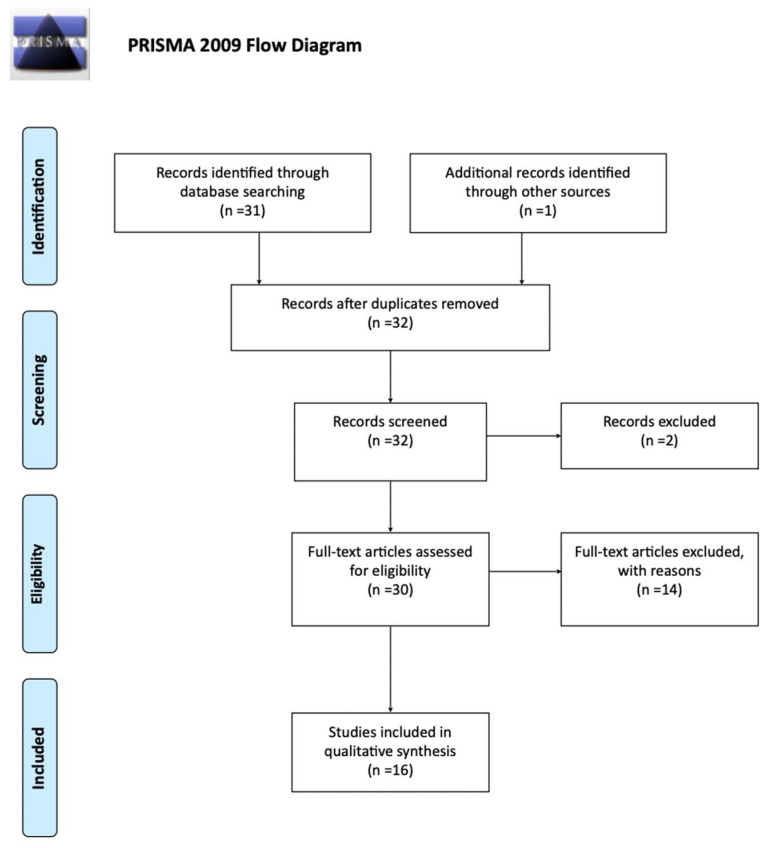
PRISMA flow chart [16].

A total of 31 studies were returned from medical databases, according to our standardized search protocol. One additional study was added after manual search [17]. Thirty-two were screened for eligibility. One was excluded due to non-English language, one due to unavailability of full-text, and the remaining fourteen studies were deemed irrelevant after full-text screening. The PRISMA flow diagram is presented in Figure 1, and schematically describes the selection process and outcome. Finally, a total of 16 studies were included in the present analysis [10,17,18,19,20,21,22,23,24,25,26,27,28,29,30,31].

The term highly differentiated follicular carcinoma of ovarian origin (HDFCO) was initially introduced by Roth et al. in 2008 after thorough investigation of the existing literature [21]. This paper included the report of one new case, the analytic presentation of another two cases, and the review of a total of fourteen patients. One of them was reported by Karselade and is independently included in the present review [19]. Another case was initially reported as malignant struma ovarii in the literature and the diagnosis was subsequently updated by Roth [21]. Therefore, this second case was included in the present review, as it is thoroughly demonstrated in the study of Roth.

### 3.2. Case Report

A 25-year-old, otherwise healthy, nulliparous woman presented to our gynecologic department due to recurrence of an ovarian mass. She had initially been subjected to excision of a mature cystic teratoma of the right ovary six years ago, which contained thyroid tissue, although lacked sufficient diagnostic criteria to qualify as a struma ovarii at the time. Four years later, a right adnexal mass with ascites and elevated CA-125 levels (1778 U/mL) was discovered. The mass was excised along with multiple peritoneal inclusion cysts and the pathology report was consistent with struma ovarii, without signs of malignant nature. During the follow-up period, MRI imaging of the abdomen revealed a mass measured at 6 × 5.7 × 4.2 cm in the remaining left ovary. In addition to that, the presence of multiple nodules was noted in the peritoneal cavity, suggestive of peritoneal infiltrations. Thyroglobulin (Tg) levels were 2047.55 ng/mL, although thyroid function tests were within normal range (TSH 1.27 μIU/mL, anti-Tg 17.23 IU/mL, anti-TPO < 28.0 U/mL).

**Figure 2:**
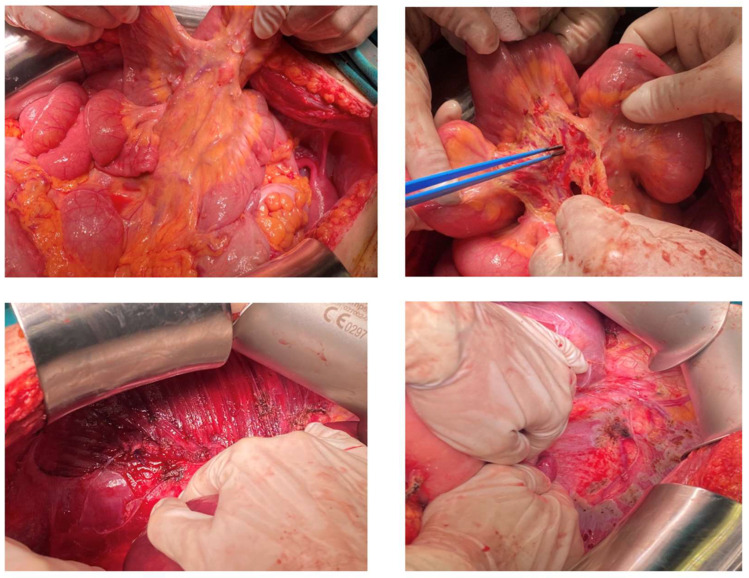
A case of a 25-year-old diagnosed with HDFCO managed at our department. Intraperitoneal lesions extended to peritoneal surfaces, including (i) the mesentery, and (ii) the subdiaphragmatic peritoneum.

The patient was referred to our gynecologic oncology department for further management. After appropriate counselling with the patient, there was a strong wish to preserve fertility. The case was discussed at an MDT, including gynecologic oncologists, pathologists, surgical oncologists, endocrinologist, and a nuclear medicine specialist. Unfortunately, the patient was referred to our gynecologic oncology department only after the recurrence. At this point, no oocytes had been cryopreserved. Before surgery, the patient received extensive counselling and all options for fertility preservation were discussed. This patient had only one ovary left, which was completely occupied by the lesions. According to the MDT’s suggestions and the patients’ wish, an attempt at cytoreduction with uterine preservation, if deemed oncologically safe, was undertaken. A laparotomy was performed with a midline incision. At initial inspection, the mass was located at the left ovary and seemed to extend to the ovarian capsule (Figure 2). The evaluation of the abdominal cavity revealed multiple, nodule-like lesions on the parietal and visceral peritoneum, on the mesentery, on the serosa of the bowel, and the sub-diaphragmatic peritoneum (Figure 2). Surgical staging was consistent with FIGO stage III B, as macroscopic lesions did not exceed 2 cm of maximal diameter. Extensive cytoreductive surgery was performed and optimal debulking was achieved with no macroscopic residual tumor. Based on our intraoperative findings, the uterus was preserved to allow for a potential pregnancy with donor oocytes in the future. The final histopathologic diagnosis was consistent with a highly differentiated follicular carcinoma arising in struma ovarii. The patient underwent total thyroidectomy and radioiodine treatment (145 mCi). Tg levels decreased to 18 ng/mL one month after the abdominal operation. Follow-up was undertaken by a multi-disciplinary team including a gynecological oncologist, pathological oncologists, endocrinologists, and a nuclear medicine specialist. During follow-up, an abdominal MRI is obtained every 4–6 months and thyroid function tests (TSH, Tg) are checked every 3 months. Patient’s latest Tg and TSH levels are within normal range, and MRI scans suggest no signs of recurrence.

### 3.3. Characteristics of the Included Patients

The sample consisted of twenty patients that fulfilled the criteria for the diagnosis of highly differentiated follicular carcinoma of ovarian origin (HDFCO), including our own case. The measured variables are collectively presented in Table 1. The mean age of the patients at diagnosis was 47.15 years (range 24–74). Mean follow-up period was 42.9 (range 4–204) months. In 92.9% of the reported cases, there was a previous gynecologic surgery ranging from cystectomy to total abdominal hysterectomy with bilateral oophorectomy. From those cases, a previous histologic diagnosis of struma ovarii was present in 62.5%, and a mature teratoma in 12.5%. In the remaining patients, the report was consistent with benign pathology other than struma ovarii, or not available. A history of HDFCO or struma peritonei was present in two patients, in both cases associated with a previous diagnosis of struma ovarii [25,30]. Interestingly, the only case reported to have parenchymal metastasis to the spleen, liver, and lung was associated with a previous diagnosis of “struma peritonei” that was surgically treated 12 years ago. This case highlights the insidious biologic behaviour of this clinical entity, which, despite its low malignant potential, has the tendency to recur and metastasize even years after initial surgery.

In a percentage as high as 57.1%, the patients were asymptomatic and the finding was incidental. Symptoms, if present, mainly included abdominal and pelvic pain and bloating. In one case, the lesions involved facial bony structures and the symptoms included temporomandibular joint pain and facial swelling. In another case with vertebral involvement, the presenting symptom was lower back pain. Ascites were present in only three cases, accounting for 42.9% among the studies that provided relevant data.

### 3.4. Characteristics of the Lesions (Location, Size)

The mean size of ovarian lesions was estimated at 8.3 cm (range 2–15). The predominant location of the lesion was the ovary in 60% of the cases. In the remaining cases, the lesions were localized in the peritoneum, the omentum, the uterus/uterine serosa, the para-aortic lymph nodes, the bowel serosa, and the epicardial lymph nodes. Extraperitoneal spread was encountered in 15% in the lungs, multiple facial bony structures, the heart, and the lumbar vertebrae.

### 3.5. Surgical Technique and Outcomes

Data concerning the surgical approach and the type of surgery are available for 17 patients. Surgery greatly varied from resection of the ovarian lesion to hysterectomy and bilateral salpingo-oophorectomy. Type of surgery included conservative management (fertility/uterine-sparing) in 35.3% (six patients), radical surgery (non-fertility/uterine-sparing) in 41.2% (seven patients), biopsies in 11.8% (two patients), and local excision after a previous history of total hysterectomy and bilateral salpingo-oophorectomy in 11.8% (two patients). Complete excision was reported in 88.3% of the cases. Mean age in the conservative surgical treatment group was 37.2 years (range 25–50), while in the radical treatment group it was 49.1 years (range 32–74). Omentectomy was performed for staging purposes in six (35.3%) and pelvic lymphadenectomy in four (23.5%) patients. In one case, lymphadenectomy was extended to the para-aortic lymph nodes. A minimally invasive approach (laparoscopy) was applied in 17.6% (3/17 patients) of the cases, and in one of them the approach was robotic-assisted.

Dobi et al. described the involvement of the sigmoid colon by the disease, which was managed by rectosigmoid resection and anastomosis. In our own case, optimal debulking included left salpingo-oophorectomy; peritoneal resection of the pelvic, paracolic, bladder, and subdiaphragmatic peritoneum; omentectomy; and appendectomy. Macroscopically enlarged peritoneal lymph nodes were also removed (Figure 2). The final pathologic report confirmed the presence of neoplastic tissue with focal stromal invasion in all specimens, apart from the lymph nodes and the appendix. Our findings highlight the potential of extensive intraperitoneal dissemination of the disease, even in the subdiaphragmatic peritoneum, which was not previously addressed.

Where relevant data were available, adjuvant therapy included thyroidectomy combined with radioactive iodine therapy in 62.5%% (10/16 cases), no additional therapy in 31.3% (5/16), and chemotherapy only in 6.25% (1 case treated with combination adriamycin 60 mg and farmorubicin 80 mg). The was one reported case with a previous history of thyroidectomy several years before the diagnosis of HDFCO. Another case described by Carey et al. had a diagnosis of concurrent thyroid carcinoma (well-differentiated papillary carcinoma), with maximal diameter of 0.5 cm, which was classified as stage PT1. The peritoneal lesions, however, were classified as HDFCO due to the distinctive histologic features and the unlikely possibility of metastasis from the thyroid carcinoma.

Concerning the prognosis, in the total of 16 patients with reported relevant outcomes, 81.25% (13/16) of the patients did not present any signs of disease during the follow-up period, 12.5% (2/16) were alive with disease, and 6.25% (1/16) had a recurrence. The recurrence pertains to a patient incidentally diagnosed with the disease during laparotomy for cholecystectomy. The treatment included excision of peritoneal disease, paraortic lymphadenectomy followed by thyroidectomy and radioiodine therapy. Τhe patient had a previous history of surgically excised struma ovarii. The disease recurred 2.5 years after the surgical treatment and was managed with repeat radioiodine therapy. The patient was living with the disease when the study was published.

In the subgroup of patients that did not receive any adjuvant therapy and were treated by surgery alone (four patients), no recurrences were reported. One patient received postoperative chemotherapy and had no evidence of disease during a follow-up period of 3 years. All cases but one were managed by radical surgical treatment.

## 4. Discussion

Highly differentiated follicular carcinoma of the ovary (HDFCO) is a rare entity described by Roth et al. in 2008 as a tumor comprised of bland thyroid tissue disseminating beyond the ovary [21]. Subsequently, the diagnosis of HDFCO is reserved for these neoplasms that present with documented extra-ovarian dissemination, which can occur even decades after the initial presentation of a benign struma ovarii [32,33]. Our case describes the recurrence of a neoplasm, initially diagnosed as benign struma ovarii, which disseminated to the contralateral ovary and to a significant extent to the visceral and parietal peritoneum of the diaphragm and the mental bursa, two years after the initial presentation, in a young nulliparous female. Due to the histologically innocuous appearance of the tumor, resembling a colloid or nodular goiter, we considered it as a highly differentiated follicular thyroid-type carcinoma as proposed by Roth [21]. The aim of the present review is to display and comment on the current evidence regarding the management and prognosis of HDFCO, including our experience with a 25-year-old female. To the best of our knowledge, this is the youngest patient reported with HDFCO up to date.

Despite the morphologically benign characteristics of the disease, dissemination of the tumor is required in order to establish the diagnosis of HDFCO [7,32]. As presented in the literature, some of the patients even presented with bone, live, lung, or heart metastases [17,21,34]. Thereafter, considering that ovarian struma is a neoplasm, and not a goiter as seen in the thyroid, its dissemination and metastatic potential to other organs reinforces the view, as cited in the last 2020 WHO classification of female genital tumors, that “the presence of peritoneal implants of well differentiated thyroid tissue in a patient with histologically benign struma ovarii known as strumosis, is now thought to represent metastasis from a highly differentiated follicular carcinoma arising in struma ovarii” [7].

The pathophysiologic mechanisms resulting in HDFCO development are not yet well understood. The hormonal changes in pregnancy have been proposed as a trigger factor enhancing thyroid cell growth, but the association lacks solid validation [35]. On a molecular level, HDFCO tissue lacks mutations commonly encountered in thyroid cancer [36,37,38]. The limited molecular analyses’ results suggest mutations of fibroblast growth factor receptor (FGFR) as the most significant epitope, influencing the tumor’s behaviour [30]. The ‘second-hit’ theory proposed by Henderson et al. is supported by the recently published work by Bao et al., based on the significant heterogeneity of ovarian and distant disease tissue [17,30]. Specifically, the FGFR4 Gly388 Arg polymorphism has been identified as a key mutation in the developing process of the tumor [30].

Consensus regarding the optimal surgical and medical management of such tumors has not been reached. There is a gap in the literature on the optimal therapeutic management of the extremely rare entity, recently classified as HDFCO. Therefore, treatment usually follows the principles of thyroid cancer treatment. Surgical treatment options vary from conservative uterine-sparing to radical surgical excisions, followed by total thyroidectomy, radioiodine treatment, and adjuvant therapy in selected cases, according to the MDT suggestion [39].

According to the results of the present review, the majority of the patients were treated by complete surgical removal of the lesions (88.3%). Interestingly in the subgroup of patients that were only subjected to surgery and did not receive any adjuvant therapy, no recurrences were observed. In two patients that presented continuing evidence of disease, surgical treatment did not involve complete excision of the lesions due to their spread (spleen, liver, lung metastasis) and localization (epicardial nodules). However, adjuvant therapy was implemented in both patients. The only case that recurred had a history of total abdominal hysterectomy and bilateral salpingo-oophorectomy, as well as a diagnosis of struma ovarii 26 years previously. The patient was treated with local excision of the intraperitoneal lesions and adjuvant thyroidectomy/radioactive therapy. However, the disease relapsed 2.5 years later. Overall, the prognosis seems favorable, as no disease-related deaths were recorded.

The lack of evidence on the optimal treatment for those patients becomes even more apparent in young females and in the context of fertility preservation, such as in our case. Despite being classified as “a thyroid neoplasm”, HDFCO involves the ovaries per se and its treatment may compromise fertility. Additionally, in many cases there is a history of a previous gynecologic surgery for struma ovarii, mature teratoma or other benign pathology, which may have already affected ovarian reserve. Based on the scarce data available, complete tumor removal is deemed a safe and reasonable strategy, although the ideal extent of surgical excision (conservative vs. radical) remains to be determined. As the effectiveness of adjuvant therapy is concerned, the data are not sufficient to make sound recommendations. The similarities shared with the thyroid gland justified the adjuvant thyroidectomy and radioactive iodine, in the absence of more solid data. In order to recommend changes in routine clinical practice, high quality studies are needed. However, the rarity of this entity precludes the conduction of large prospective studies. In this context, data derived from systematic reviews of the literature of published cases represent the best available evidence to assist clinical practice. Further research is needed to shed light on the extremely rare pathology of HDFCO.

### Strengths and Limitations

This study was conducted though meticulous review of the current literature, which was carried out simultaneously by three independent reviewers. Any discrepancies were unanimously resolved by the reviewers. However, there were several limitations inherent to this study. First of all, cases falsely assigned the diagnosis of malignant struma ovarii, which in fact represented highly differentiated follicular carcinoma of ovarian origin, are not included. Hence, relevant cases may have been omitted. We decided to include only cases that were described in a way that the diagnosis of HDFCO cannot be questioned. Another limitation is that there are only limited studies in the literature describing cases of HDFCO and the majority represents scarce case reports. No other original study design is available. Nevertheless, the follow-up is limited or not stated in many studies. As a consequence, it is impossible to draw any safe conclusions for the optimal management, as long as information regarding overall survival, disease- free survival, and oncologic safety are unavailable.

## 5. Conclusions

Data are not sufficient to draw any sound conclusions regarding the optimal balance between radical excision of the tumor and minimization of treatment consequences. Questions regarding the radicality of surgery and its impact on prognosis, the need of adjuvant therapy (thyroidectomy and radioactive iodine therapy), and the prognostic value of a classification system remain unaddressed. For these reasons, the significance of the establishment of a homogenous terminology to allow for precise and efficient documentation and data collection is underlined, in order to facilitate further research.

## Figures and Tables

**Table 1:** Patients’ characteristics and measured outcomes.

Author; Year	Age (Years)	Previous Gynecologic Surgery	Diagnosis	Clinical Presentation	Lesion Location	Max Diameter of Ovarian Lesion	Ascites	Surgical Management	Thyroidectomy	RAI	Chemo-therapy	Follow-Up (Months)	Recurrence	Diagnosis
Balasch et al., 1993 [18]	36	RO	SO	Incidental finding during IVF protocol	Left ovary, adnexa (Rt), omentum, large bowel	8 cm	NO	LSO and local excision	NA (recommended)	NA	NO	NA	NA	Metastatic ovarian strumosis
Karselade; 1994 [19]	49	RSO	Simple cyst	Incidental finding during laparotomy	Left ovary; omentum	5 cm	YES	TAH + LSO	NO	NO	YES	36	NED	Peritoneal strumosis
Brogsitter; 2004 [20]	50	NA	NA	NA	Right ovary, peritoneum	NA	NA	RSO and local excision	YES	YES	NO	6	NED	Benign strumosis
Roth et al., 2008 [21]	58	RSO; TAH + LSO	Ectopic pregnancy; SO	Incidental finding during laparotomy	Peritoneum, omentum, para-aortic lymph nodes	NA	NO	Excision of peritoneal/omental nodules and paraaortic lymph node, appendectomy, liver biopsy	YES	YES	NO	96	YES/2.5 y Repeat RAI LWD	HDFCO
	50	NA	NA	Incidental finding during laparotomy	Right ovary, omentum, bladder serosa, Douglas pouch	15 cm	NA	STAH + RSO, omentectomy and excision of peritoneal nodules	YES	YES	NO	72	NED	MSO (updated by Roth et al. as HDFCO)
Kim et al., 2009 [22]	49	LO	Benign tumor (not specified)	Abdominal discomfort, palpitations, flush	Right ovary, omentum, bladder dome, rectosigmoid mesocolon, Douglas space	10 cm	YES (300 mL)	TAH—right ovarian tumor excision, implant excision, bilateral pelvic lymphadenectomy	YES	YES	NO	15	NED	Peritoneal strumosis
Sibio et al., 2010 [23]	74	NO	NA	Pelvic pain	Right ovary, peritoneum	13 cm	NA	TAH + BSO, implant excision, locoregional lymphadenectomy	Thyroidectomy (12 years)	NO	NO	84	NED	Brenner’s tumor and malignant struma ovarii with benign strumosis
Carey et al., 2014 [24]	70	TAH + BSO	Report not available	Asymptomatic	Peritoneal and epicardial nodule	NA	NA	Local excision and partial omentectomy (laparoscopic)	YES	YES	NO	4	LWD	Extraovarian struma ovarii
Ranade et al., 2014 [25]	55	YES (×2) type of surgery NA	Struma ovarii; Struma peritonei	NA	Adnexa, peritoneal nodules, liver, spleen, lungs	NA	NA	Biopsy of peritoneal nodules	YES	YES	NO	NA	LWD	HDFCO
Wei et al., 2015 [10]	35	NA	NA	NA	Right ovary, fallopian tube, urinary bladder, pelvic peritoneum	NA	NA	Excision of the mass and peritoneal nodules	NA	NA	NA	204	NED	HDFCO
Riggs et al., 2018 [26]	32	RSO; LSO	Mature teratoma (ruptured); simple cyst	Abdominal pain	Uterine serosa (anterior and posterior peritoneal reflections)	NA	NA	Modified hysterectomy and complete pelvic peritoneal resection (robotic-assisted laparoscopy)	No (thyroid preservation)	NO	NO	12	NED	HDFCO
Dobi et al., 2019 [27]	52	NA	N	Abdominal bloating	Uterus, bowel serosa, omentum	12.5 cm	YES	TAH + BSO, omentectomy, rectosigmoid resection and anastomosis, left pelvic and common iliac lymphadenectomy	NO	NO	NO	12	NED	HDFCO
Prentice et al., 2020 [28]	33	Ovarian cystectomy	SO (piecemeal extraction)	Incidental finding during follow-up	Peritoneum, pelvic sidewall, pararectal spaces, uterosacral ligaments	NA	NA	N/A	YES	YES	NO	NA	NED	HDFCO
Henderson, 2020 [17]	71	NA	Pelvic dermoid	Right temporomandibular joint discomfort and facial swelling	Face, bones, liver, heart	NA	NA	Biopsy of facial and heart lesions	YES	YES	NO	18	NED	Multifocal metastatic struma ovarii
	58	NA	SO	NA	Not specified para-aortic lymph node	NA	NA	NA	NA	NA	NA	NA	NA	Extraovarian struma ovarii
	31	RSO	SO	NA	Not specified peritoneum	8 cm	NA	NA	NA	NA	NA	NA	NA	Extraovarian struma ovarii
Li et al., 2021 [29]	39	Bilateral ovarian cystectomy (LAP)	SO (intact extraction)	Incidental finding during follow-up	Ovary, fallopian tube, uterus, urinary bladder, pelvic wall, sigmoid colon	NA	NO	Open surgery (during caesarian section), resection of ovarian mass, and local lesion excision	NO	NO	NO	NA	NA	Peritoneal strumosis
Bao et al., 2022 [30]	38	Ovarian cystectomy Rt; ovarian cystectomy Rt; ovarian cystectomy Rt and lesion excision	OMCT; SO; HDFCO	NA	Ovary, peritoneum, rectus abdominis, rectum, para-aortic lymph nodes	2 cm	NA	TLH + BSO, omentectomy, pelvic and para-aortic lymphadenectomy, local excision	YES	YES	NO	10	NED	HDFCO
Asaturova et al., 2022 [31]	38	Ovarian cystectomy during CS	SO	Lower back pain	Ovary, lumbar vertebra, omentum, Sigmoid	3.5 cm	NA	Laparoscopic LO, omentectomy and local excision	NO (patient’s refusal)	NO	NO	17	NED	HDFCO
Giovannopoulou et al., 2022	25	Ovarian cystectomy; RSO	Mature teratoma; struma ovarii	Asymptomatic	Left ovary, peritoneum, mesentery, bowel serosa, subdiaphragmatic peritoneum	6 cm	NO	Optimal debulking uterine preservation, LSO, omentectomy, Pelvic, paracolic, subdiaphragmatic, and bladder peritoneum, appendectomy	YES	YES	NO	15	NED	HDFCO
Total	47.15 (25–74)	92.9% previous gynecologic surgery	62.5 % previous diagnosis of SO	57.1% asymptomatic	60% ovarian 15% extraperitoneal disease	8.3 (2–15) cm	42.9%	35.3% uterine-sparing, 41.2% radical, 11.8% biopsies, 11.8% local excision (previous TAH + BSLO)	62.5%% combined thyroidectomy and RA, 31.3% no additional therapy, 6.3% chemotherapy			42.9 months (4–204)	81.25% NED 12.5% LWD 6.25% recurrence	50% HDFCO 50% other terminology

RAI: radioiodine therapy, F-UP: follow-up, RO: right oophorectomy, RSO: right salpingo-oophorectomy, ROV: right ovary, LOV: left ovary, IVF: in vitro fertilisation, LSO: left salpingo-oophorectomy, TAH: total abdominal hysterectomy, NED: no evidence of disease, LWD: living with disease, SO: struma ovarii, MSO: malignant struma ovarii, STAH: subtotal abdominal hysterectomy, BSO: bilateral salpingo-oophorectomy, TLH: total laparoscopic hysterectomy, CS: caesarean section, NA: non-applicable/not available.

## Data Availability

All data are available upon request to the corresponding author.
